# Predicting response to immune checkpoint inhibitor plus chemotherapy in EGFR-mutant lung adenocarcinoma following first-generation TKI resistance: a multicenter deep learning study

**DOI:** 10.3389/fimmu.2026.1760264

**Published:** 2026-07-17

**Authors:** Shuai Qie, Yasong Shi, Jingyun Li, Sicong Jia, Xiaoping Yin

**Affiliations:** Department of Radiation Oncology, Hebei University Affiliated Hospital, Baoding, Hebei, China

**Keywords:** computed tomography (CT), deep learning, EGFR-mutant lung adenocarcinoma, immune checkpoint inhibitor, radiomics, treatment response prediction

## Abstract

**Background:**

Patients with epidermal growth factor receptor (EGFR)-mutant lung adenocarcinoma who develop resistance to first-generation tyrosine kinase inhibitors (TKIs) without a T790M mutation face a therapeutic dilemma with limited and suboptimal options.

**Methods:**

In this multicenter retrospective study, the final analyzable modeling cohort included 490 patients with complete eligible CT imaging and outcome labels, comprising a training cohort of 326 patients, validation cohort 1 of 70 patients, and validation cohort 2 of 94 patients. Model performance was evaluated using AUC, decision curve analysis, and PFS stratification analyses.

**Results:**

The 2.5D axial model achieved AUCs of 0.885, 0.819, and 0.863 in the training cohort, validation cohort 1, and validation cohort 2, respectively, based on the locked source prediction files.

**Conclusion:**

A CT-based 2.5D deep learning model showed promising performance for treatment-response prediction after EGFR-TKI resistance, but prospective validation and clinical-variable benchmarking remain necessary before clinical implementation.

## 1 Introduction

Lung cancer remains the leading cause of cancer-related mortality globally, with EGFR mutations being the most prevalent oncogenic drivers in adenocarcinoma (1). While EGFR-TKIs like Osimertinib have revolutionized first-line treatment, acquired resistance invariably develops, creating a critical therapeutic challenge with limited and suboptimal subsequent options (2, 3).

ICIs have reshaped the landscape of advanced non-small cell lung cancer (NSCLC) without actionable drivers. However, their efficacy in EGFR-mutant NSCLC, particularly after TKI failure, remains modest and unpredictable, compounded by the risk of hyperprogression (4, 5). Current biomarkers for guiding ICI therapy in this setting are inadequate. PD-L1 tumor proportion score demonstrates limited predictive value and significant heterogeneity (6), while obtaining post-resistance tumor tissue for advanced profiling (e.g., tumor mutational burden, tumor microenvironment analysis) is often impractical due to its invasive nature, sampling bias, and patient ineligibility (7). Consequently, there is an urgent, unmet need for a non-invasive tool to predict ICI benefit in this patient population.

Deep learning has emerged as a powerful technique for decoding complex information from medical images (8, 9). We hypothesize that the dynamic biological evolution of tumors during the development of TKI resistance—including alterations in the tumor immune microenvironment—imparts discernible, albeit subtle, signatures on routine CT scans. These latent imaging phenotypes may encapsulate critical information predictive of subsequent response to immunotherapy (10, 11).

Existing studies are primarily limited to predicting static genetic alterations using radiomics or deep learning, as consolidated by a systematic review on EGFR mutation prediction in NSCLC (12, 13) or focus on ICI response in TKI-naïve populations (14–16). A significant gap exists in leveraging advanced deep learning to directly predict ICI efficacy specifically in the post-TKI resistance setting. Furthermore, many approaches rely on multi-step, handcrafted feature engineering, which may introduce bias and fail to capture the most salient patterns.

To address this, we propose that an end-to-end deep learning model can directly decode these complex visual patterns from baseline CT images acquired after EGFR-TKI resistance to predict immunotherapy outcomes accurately. This study aims to develop and validate such a model using a large, multi-center retrospective cohort. Our framework takes post-resistance, pre-immunotherapy CT images as direct input to predict objective response to ICI-chemotherapy and to evaluate model-derived PFS risk stratification.

## 2 Materials and methods

### 2.1 Study participants

**Figure 1:**
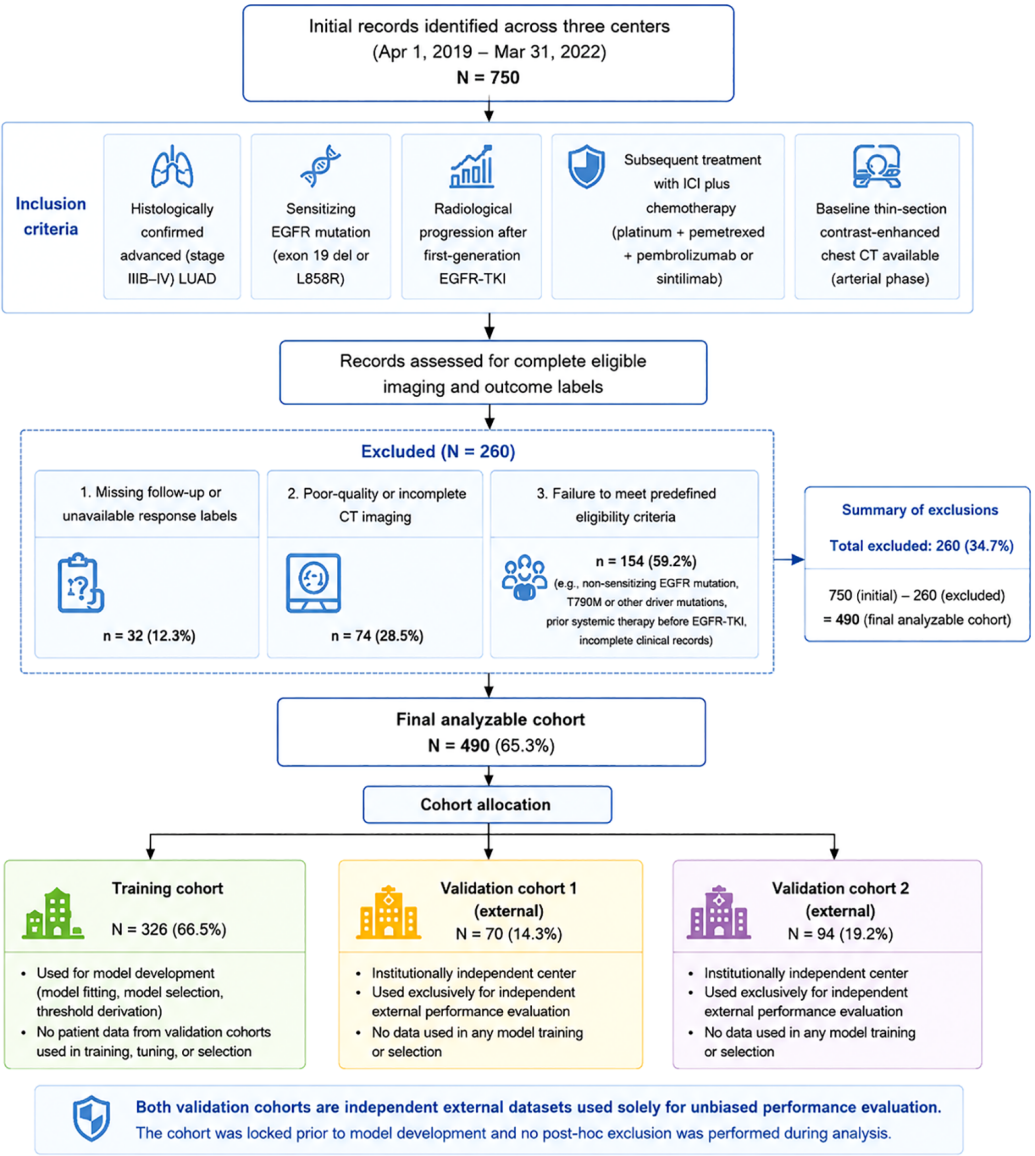
Study participant flowchart.

The patient selection process is shown in **Figure 1**. The initially identified screening cohort consisted of 750 patients from three centers. After application of predefined inclusion and exclusion criteria, 260 patients were excluded, resulting in a final analyzable cohort of 490 patients with complete eligible CT imaging and treatment response labels. The reasons for exclusion were as follows: (1) missing follow-up or unavailable treatment response labels (n = 32), (2) poor-quality or incomplete CT imaging unsuitable for quantitative analysis (n = 74), and (3) failure to meet predefined eligibility criteria, including absence of sensitizing EGFR mutation, presence of T790M or other driver mutations, prior systemic therapy before EGFR-TKI initiation, or incomplete clinical records (n = 154). To ensure transparency and consistency, cohort nomenclature was standardized throughout the revised manuscript, figures, and **Supplementary Materials**. The final analyzable cohort was divided into a training cohort (n = 326), validation cohort 1 (n = 70), and validation cohort 2 (n = 94). The training cohort was used for model development, including model fitting, model selection, and threshold derivation. Validation cohort 1 and validation cohort 2 were derived from institutionally independent centers and therefore served as two independent external validation cohorts. Both validation cohorts were used exclusively for independent performance evaluation. No data from either validation cohort were used for model training, hyperparameter tuning, threshold selection, threshold derivation, or model selection.

### 2.2 Molecular profiling and treatment pathway justification

To ensure the enrolled population was representative of patients for whom ICI-chemotherapy is a standard option, all patients were required to have undergone molecular profiling following resistance to first-generation EGFR-TKIs. Patients were included only if there was no evidence of the EGFR T790M resistance mutation in their medical records, making them ineligible for third-generation EGFR-TKIs (e.g., Osimertinib). The exclusion of other driver mutations was also verified as per the exclusion criteria. Molecular profiling was primarily performed using tissue-based next-generation sequencing (NGS). When tissue was unavailable or insufficient, liquid biopsy (circulating tumor DNA analysis) was used as an alternative. This stringent patient selection guarantees that the study cohort accurately reflects the clinical scenario of patients with EGFR-mutant lung adenocarcinoma who have exhausted targeted therapy options and are candidates for ICI-chemotherapy.

### 2.3 Clinical and pathological data collection

Comprehensive clinical and pathological data were retrospectively collected for all enrolled patients. The variables included age, sex, smoking history, Eastern Cooperative Oncology Group (ECOG) performance status, pretreatment clinical stage, tumor location, specific EGFR mutation subtype (exon 19 deletion or L858R), treatment regimen, and subsequent systemic therapy after ICI-chemotherapy where available. PD-L1 tumor proportion score was also collected when it had been tested in routine clinical care; because PD-L1 testing was not uniformly available across all participating centers, PD-L1-based analyses were considered exploratory subset analyses. Staging was performed according to the 8th edition of the TNM classification system established by the International Association for the Study of Lung Cancer (IASLC).

### 2.4 Pretreatment evaluation

Prior to the initiation of immune-checkpoint inhibitor combined with chemotherapy (ICI-chemotherapy), a comprehensive pretreatment evaluation was performed for all patients to ensure they were suitable candidates and to establish a baseline for response assessment. ECOG performance status was recorded before treatment initiation. Radiographic tumor assessment was performed within 4 weeks before treatment commencement using contrast-enhanced CT of the chest, abdomen, and pelvis. Brain imaging with magnetic resonance imaging (MRI) or CT was obligatory. Laboratory tests included a complete blood count, comprehensive metabolic panel, and screening for hepatitis B and C serology. This standardized workup ensured adequate organ function and no uncontrolled comorbidities.

### 2.5 Treatment regimens

Following confirmed disease progression after first-generation EGFR-TKI therapy, enrolled patients received ICI plus chemotherapy as subsequent systemic therapy. Chemotherapy regimens included platinum-based combinations with pemetrexed, paclitaxel, or other physician-selected regimens, and immune checkpoint inhibitors mainly included PD-1 or PD-L1 inhibitors. Treatment was administered according to institutional practice and contemporary clinical guidelines.

### 2.6 Response assessment and follow-up

Tumor response was evaluated by board-certified radiologists according to the Response Evaluation Criteria in Solid Tumors (RECIST) version 1.1. The radiologists were blinded to model predictions and cohort allocation during response assessment. Radiographic assessments were conducted every two cycles, approximately every 6 weeks, during the first 6 months of ICI-chemotherapy and every 3 months thereafter until disease progression, death, or the last available follow-up.

The primary classification endpoint for model development was objective response after ICI-chemotherapy. Response was defined as the best overall response achieved during ICI-chemotherapy and was classified as complete response (CR), partial response (PR), stable disease (SD), or progressive disease (PD) according to RECIST 1.1. Objective response rate (ORR) was defined as the proportion of patients who achieved CR or PR. In the locked label file, label = 1 denoted response-positive disease, corresponding to CR or PR, whereas label = 0 denoted response-negative disease, corresponding to SD or PD.

Progression-free survival (PFS) was used as the time-to-event endpoint for survival stratification. PFS was defined as the time interval from initiation of ICI-chemotherapy to the first documented radiological progression or death from any cause. Patients without progression or death were censored at the date of their last radiographic assessment. Overall survival was not included in the survival analyses reported in this manuscript because it may be substantially confounded by subsequent lines of therapy after disease progression.

### 2.7 CT acquisition and preprocessing

**Figure 2:**
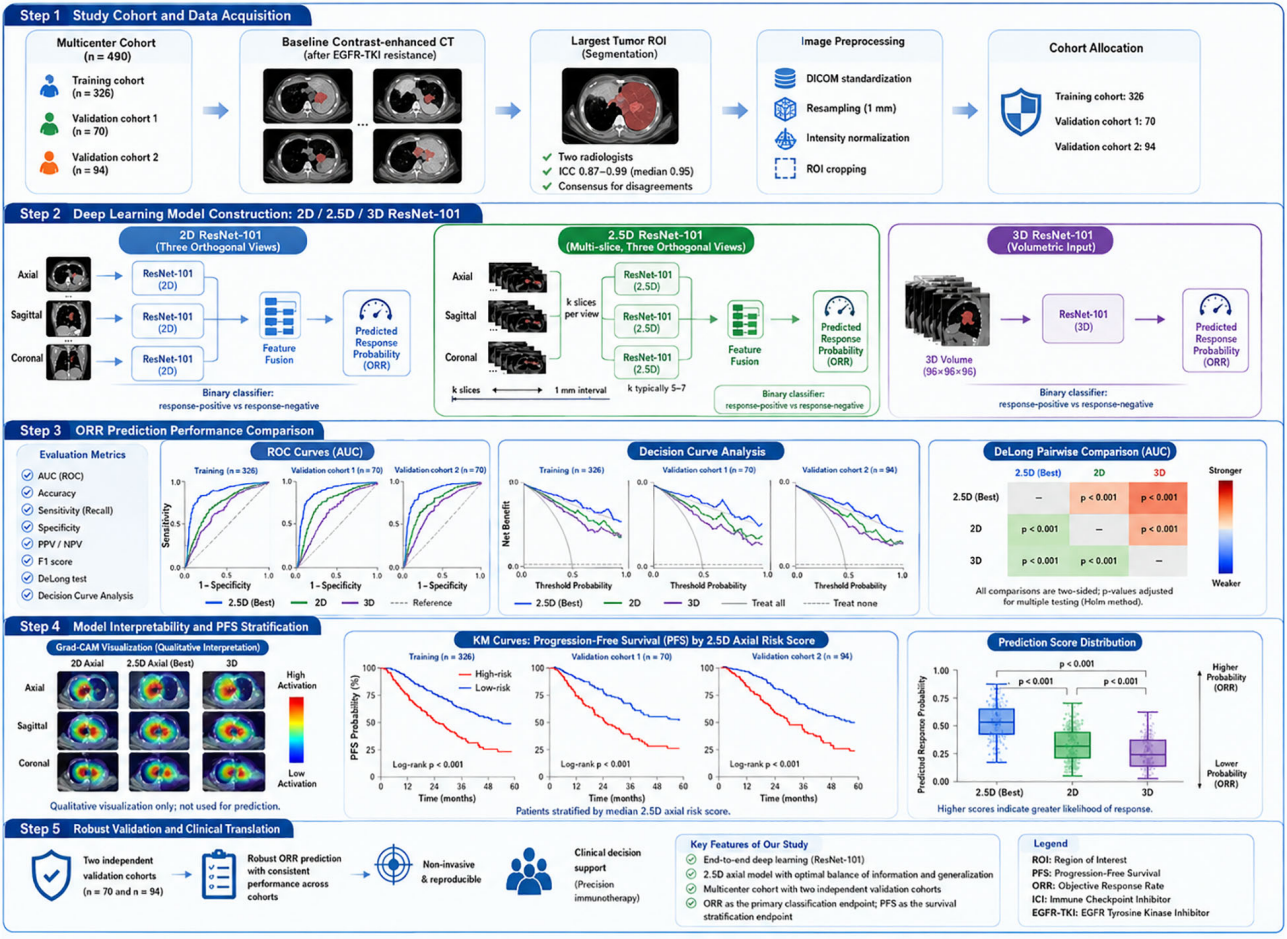
Schematic overview of the deep learning study workflow.

**Figure 2** depicts the schema of the present study. Lesion segmentation was performed independently by two observers who were blinded to treatment outcomes. Interobserver reproducibility was assessed using the available 30-case subset with paired feature extraction files. ICC values ranged from 0.973 to 1.000, with a median ICC of 0.998, supporting excellent reproducibility. Cases with segmentation disagreement were resolved by consensus review.

### 2.8 2D Resnet-101 model development

A two-dimensional (2D) ResNet-101 model was developed to predict objective response to ICI-chemotherapy using CT images from three orthogonal views: axial, sagittal, and coronal. For each view, the tumor-centered slice with the largest tumor area was selected according to the segmented region of interest (ROI). The ROI patch was cropped and resized to 256 × 256 pixels before being used as model input.

Three independent view-specific ResNet-101 models were constructed for the axial, sagittal, and coronal views. The ResNet-101 backbone was initialized using ImageNet-pretrained weights and fine-tuned on the training cohort. Data augmentation was applied during training, including random horizontal and vertical flipping and center cropping. The final fully connected layer was replaced with a task-specific binary classification layer, and the model output represented the predicted probability of response-positive disease. Binary cross-entropy loss was used for the ORR classification task. Model parameters were optimized using the Adam optimizer with a batch size of 64, and dropout regularization was applied to reduce overfitting.

After training the three view-specific models, feature representations from the penultimate layers were fused and passed through a final classifier to generate the overall 2D prediction score for objective response.

### 2.9 2.5D Resnet-101 model development

In this study, the term 2.5D refers to a multi-channel pseudo-volumetric approach in which five adjacent CT slices are stacked as input channels and processed by a 2D convolutional neural network backbone. This strategy was designed to incorporate limited through-plane spatial context while retaining the computational efficiency and transfer-learning advantages of 2D CNNs.

For each orthogonal view, including axial, sagittal, and coronal views, the central slice with the largest tumor area was first identified. Two adjacent slices on each side of the central slice were then selected at 1-mm intervals, resulting in five consecutive slices per view. These five slices were stacked as five input channels to construct the 2.5D input. For each view, the minimum bounding rectangle encompassing the tumor region across all five slices was defined as the ROI, cropped, and resized to 256 × 256 pixels.

Three independent 2.5D ResNet-101 models were trained for the axial, sagittal, and coronal views. The ResNet-101 backbone was initialized using ImageNet-pretrained weights and fine-tuned on the training cohort. The first convolutional layer was modified to accommodate five-channel input. The remaining network architecture followed the standard ResNet-101 design with residual skip connections. Data augmentation strategies were consistent with those used for the 2D models, including random flipping and center cropping.

The final fully connected layer was replaced with a binary classification layer, and the model was trained to output the predicted probability of response-positive disease after ICI-chemotherapy. Binary cross-entropy loss was used as the classification loss. Model optimization was performed using the Adam optimizer with an initial learning rate of 0.001 and a batch size of 64. Dropout regularization was incorporated to reduce overfitting. The 2.5D model therefore learned an enriched tumor representation from adjacent slices and multiple anatomical planes for end-to-end prediction of objective response.

### 2.10 3D Resnet-101 model development

To compare the 2D and 2.5D approaches with a volumetric deep learning strategy, a three-dimensional (3D) ResNet-101 model was also developed. In 3D data processing, tumor ROIs were encapsulated within bounding cubes and uniformly resampled to 96 × 96 × 96 voxels using linear interpolation. Data augmentation was applied by mirror flipping along the X, Y, and Z axes.

Because directly transferable ImageNet-pretrained weights are not readily available for standard 3D medical imaging models, the 3D ResNet-101 model was initialized from scratch and trained on the training cohort. The final fully connected layer was replaced with a binary classification layer to predict response-positive disease after ICI-chemotherapy. Binary cross-entropy loss was used for model training. The model was trained for 200 epochs using the Adam optimizer.

The 3D model output represented the predicted probability of objective response. No survival-specific loss function or Cox proportional hazards layer was used in the deep learning models. PFS was evaluated separately as a time-to-event endpoint for risk stratification based on the model output score, as described in the statistical analysis section.

### 2.11 Model training, class imbalance, and threshold selection

All model training, hyperparameter selection, and threshold derivation were performed using the training cohort only. Validation cohort 1 and validation cohort 2 were not used for model training, hyperparameter tuning, threshold selection, threshold derivation, or model selection.

In the training cohort, the response-positive and response-negative groups included 118 and 208 patients, respectively, indicating moderate class imbalance. No oversampling, undersampling, or class weighting strategy was applied. The class distribution was reported to support transparent interpretation of threshold-dependent performance metrics.

For each model, the classification threshold was determined in the training cohort using the Youden index and then applied unchanged to validation cohort 1 and validation cohort 2. Thresholds were not re-optimized in either validation cohort.

### 2.12 Statistical analysis

Statistical analyses were performed using SPSS 20.0 and Python 3.9.1. Continuous variables were compared using t-tests or Mann-Whitney U tests, and categorical variables were analyzed using chi-square or Fisher’s exact tests. Model performance was evaluated using AUC, accuracy, sensitivity, specificity, PPV, NPV, and F1 score. For each architecture, the classification threshold was selected in the training cohort using the Youden index and then applied unchanged to validation cohort 1 and validation cohort 2. PFS was analyzed as the time-to-event endpoint for survival stratification.

Due to incomplete availability and limited harmonization of some clinical variables across centers, formal clinical-only and combined clinical-imaging predictive models were not constructed in the present study. This issue is acknowledged as a limitation and should be addressed in future prospective studies.

## 3 Results

### 3.1 Clinical characteristics

The final analyzable cohort included 490 patients, comprising 326 patients in the training cohort, 70 patients in validation cohort 1, and 94 patients in validation cohort 2. According to RECIST-defined objective response, the response-positive/response-negative distribution was 118/208 in the training cohort, 20/50 in validation cohort 1, and 29/65 in validation cohort 2, corresponding to ORRs of 36.2%, 28.6%, and 30.9%, respectively. The overall ORR was 34.1% among the 490 patients.

Baseline clinical characteristics across the three cohorts are summarized in **Table 1**. Most baseline variables, including age, sex, T stage, N stage, primary tumor site, smoking status, comorbidities, EGFR subtype, metastatic status, chemotherapy regimen, and immune checkpoint inhibitor category, were generally comparable across cohorts. However, drinking status and PFS event distribution differed significantly among cohorts and were therefore considered when interpreting survival-related analyses. Baseline characteristics stratified by treatment response status are provided in **Supplementary Table 1**.

**Table 1:** Baseline characteristics across the training cohort and two validation cohorts.

Characteristic	ALL (N = 490)	Training cohort (N = 326)	Validation cohort 1(N = 70)	Validation cohort 2 (N = 94)	P value
Sex					0.497
Female	306 (62.45%)	206 (63.19%)	46 (65.71%)	54 (57.45%)	
Male	184 (37.55%)	120 (36.81%)	24 (34.29%)	40 (42.55%)	
Age	62 (55-68)	62 (55-69)	62 (55-67)	61.50 (56-66)	0.711
T stage					0.410
T1	100 (20.41%)	69 (21.17%)	12 (17.14%)	19 (20.21%)	
T2	176 (35.92%)	106 (32.52%)	31 (44.29%)	39 (41.49%)	
T3	72 (14.69%)	48 (14.72%)	11 (15.71%)	13 (13.83%)	
T4	142 (28.98%)	103 (31.60%)	16 (22.86%)	23 (24.47%)	
N stage					0.378
N0	93 (18.98%)	71 (21.78%)	7 (10.00%)	15 (15.96%)	
N1	39 (7.96%)	24 (7.36%)	7 (10.00%)	8 (8.51%)	
N2	195 (39.80%)	126 (38.65%)	32 (45.71%)	37 (39.36%)	
N3	163 (33.27%)	105 (32.21%)	24 (34.29%)	34 (36.17%)	
Chemotherapy regimen					0.743
Pemetrexed	297 (60.61%)	191 (58.59%)	44 (62.86%)	62 (65.96%)	
Paclitaxel	107 (21.84%)	76 (23.31%)	14 (20.00%)	17 (18.09%)	
other	86 (17.55%)	59 (18.10%)	12 (17.14%)	15 (15.96%)	
ECOG					0.416
0-1	434 (88.57%)	285 (87.42%)	65 (92.86%)	84 (89.36%)	
2	56 (11.43%)	41 (12.58%)	5 (7.14%)	10 (10.64%)	
Primary site					0.396
Left	209 (42.65%)	136 (41.72%)	35 (50.00%)	38 (40.43%)	
Right	281 (57.35%)	190 (58.28%)	35 (50.00%)	56 (59.57%)	
Diabetes					0.243
No	422 (86.12%)	277 (84.97%)	59 (84.29%)	86 (91.49%)	
Yes	68 (13.88%)	49 (15.03%)	11 (15.71%)	8 (8.51%)	
Hypertension					0.433
No	307 (62.65%)	204 (62.58%)	40 (57.14%)	63 (67.02%)	
Yes	183 (37.35%)	122 (37.42%)	30 (42.86%)	31 (32.98%)	
Heart disease					0.241
No	381 (77.76%)	258 (79.14%)	49 (70.00%)	74 (78.72%)	
Yes	109 (22.24%)	68 (20.86%)	21 (30.00%)	20 (21.28%)	
Smoking					0.413
No	360 (73.47%)	243 (74.54%)	53 (75.71%)	64 (68.09%)	
Yes	130 (26.53%)	83 (25.46%)	17 (24.29%)	30 (31.91%)	
Drinking					<0.001
No	214 (43.67%)	86 (26.38%)	63 (90.00%)	65 (69.15%)	
Yes	276 (56.33%)	240 (73.62%)	7 (10.00%)	29 (30.85%)	
BMI	23.90 (22.20-25.40)	23.90 (22.00;25.40)	23.90 (22.68;25.12)	23.90 (22.80-25.50)	0.582
ORR					0.362
Non-response	323 (65.92%)	208 (63.80%)	50 (71.43%)	65 (69.15%)	
Response	167 (34.08%)	118 (36.20%)	20 (28.57%)	29 (30.85%)	
EGFR subtype					0.892
19del	186 (37.96%)	124 (38.04%)	25 (35.71%)	37 (39.36%)	
21 L858R	304 (62.04%)	202 (61.96%)	45 (64.29%)	57 (60.64%)	
Brain metastasis					0.615
No	318 (64.90%)	208 (63.80%)	49 (70.00%)	61 (64.89%)	
Yes	172 (35.10%)	118 (36.20%)	21 (30.00%)	33 (35.11%)	
Liver metastasis					0.744
No	443 (90.41%)	293 (89.88%)	65 (92.86%)	85 (90.43%)	
Yes	47 (9.59%)	33 (10.12%)	5 (7.14%)	9 (9.57%)	
Bone metastasis					0.704
No	256 (52.24%)	166 (50.92%)	38 (54.29%)	52 (55.32%)	
Yes	234 (47.76%)	160 (49.08%)	32 (45.71%)	42 (44.68%)	
ICI					0.931
PD-L1 inhibitor	17 (3.47%)	11 (3.37%)	2 (2.86%)	4 (4.26%)	
PD-1 inhibitor	473 (96.53%)	315 (96.63%)	68 (97.14%)	90 (95.74%)	
median PFS	6.7(3.82-11.8)	6.90 (4.03-12.40)	6.70 (3.75-10.90)	5.70 (3.30-11.12)	0.168
PFS event					0.028
No	100 (20.41%)	74 (22.70%)	6 (8.57%)	20 (21.28%)	
Yes	390 (79.59%)	252 (77.30%)	64 (91.43%)	74 (78.72%)	

PD-L1 expression status was not uniformly available across all participating centers and was therefore not included in primary model development.

### 3.2 Performance comparison of 2D, 2.5D, and 3D deep learning models

**Figure 3:**
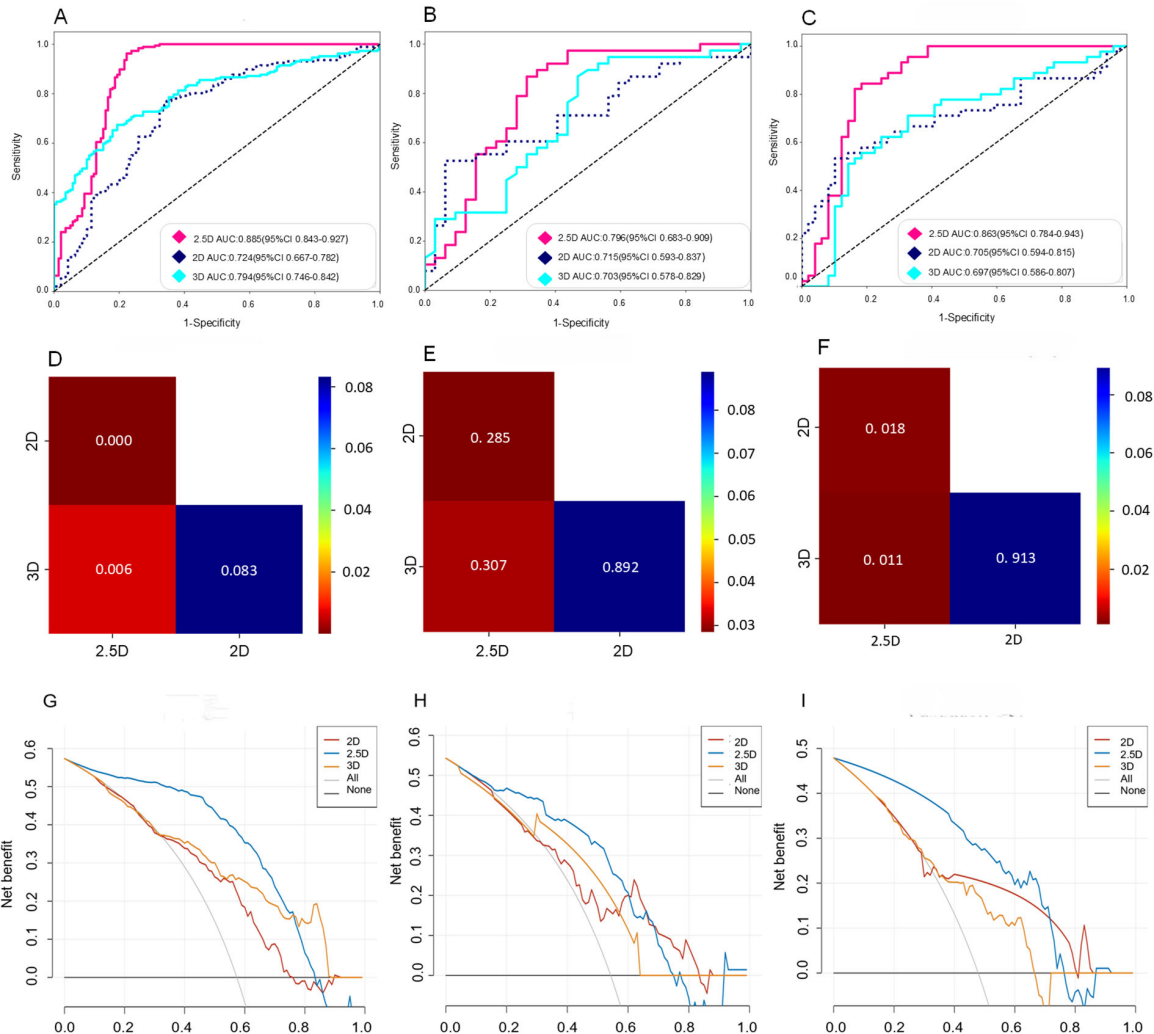
Performance comparison of the 2D, 2.5D, and 3D deep learning models. **(A–C)** Receiver operating characteristic curves for the training cohort, validation cohort 1, and validation cohort 2, respectively. **(D–F)** DeLong pairwise comparison heatmaps for the training cohort, validation cohort 1, and validation cohort 2, respectively. **(G–I)** Decision curve analysis for the training cohort, validation cohort 1, and validation cohort 2, respectively.

A comprehensive comparison of model architectures is summarized in **Figure 3** and **Supplementary Tables S3**-**S5**. Among the evaluated architectures, the 2.5D axial ResNet-101 model showed the most stable discrimination across the training and validation cohorts, achieving AUCs of 0.885 in the training cohort, 0.819 in validation cohort 1, and 0.863 in validation cohort 2, based on the final locked prediction files.

Threshold-dependent performance metrics, including accuracy, sensitivity, specificity, PPV, NPV, and F1 score, were recalculated using thresholds derived from the training cohort only. These thresholds were then applied unchanged to validation cohort 1 and validation cohort 2 to avoid optimistic bias from validation-cohort-specific threshold optimization.

Pairwise AUC comparisons were assessed using DeLong tests with correction for multiple comparisons. Therefore, the revised text avoids overstating statistical superiority when adjusted P values were not significant. The 2.5D axial model is described as the numerically best and most stable model across cohorts, whereas statistical superiority is claimed only for comparisons that remained significant after multiplicity correction.

### 3.3 PFS stratification using the 2.5D axial ResNet-101 model

The output of the 2.5D axial model was further evaluated for PFS risk stratification. Because the model was trained to predict response-positive disease, the predicted response probability was converted into a risk score, defined as 1 − response probability, so that a higher score indicated a higher predicted risk of non-response. Patients were stratified into high-risk and low-risk groups using a cutoff derived from the training cohort, and the same cutoff was applied unchanged to validation cohort 1 and validation cohort 2.

**Figure 4:**
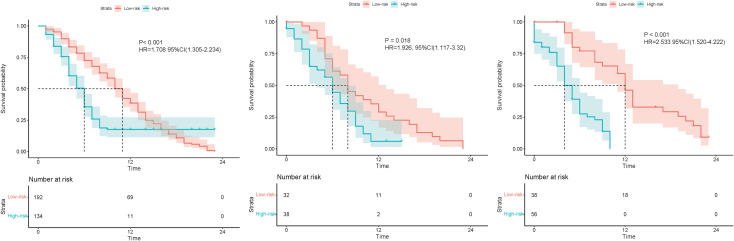
Progression-free survival stratification by the 2.5D axial ResNet-101 model. Kaplan–Meier curves comparing PFS between high-risk and low-risk groups in the training cohort **(A)**, validation cohort 1 **(B)**, and validation cohort 2 **(C)**.

Kaplan–Meier analysis demonstrated significant PFS stratification between the high-risk and low-risk groups across all three cohorts (**Figure 4**). In the training cohort, patients in the high-risk group had significantly shorter PFS than those in the low-risk group [median PFS: 6 months vs 11 months; HR = 1.708, 95% CI: 1.305-2.234, log-rank P < 0.001]. Similar PFS separation was observed in validation cohort 1 [median PFS: 6 months vs 8 months; HR = 1.926, 95% CI: 1.117-3.320, log-rank P = 0.018] and validation cohort 2 [median PFS: 4 months vs 12 months; HR = 2.533, 95% CI: 1.52-4.222, log-rank P < 0.001].

Multivariable Cox proportional hazards regression further confirmed that the 2.5D risk group was associated with PFS after adjustment for available clinical variables in the training cohort, validation cohort 1, and validation cohort 2 (**Supplementary Tables S7**-**S9**).

### 3.4 Interpretability analysis of the deep learning models

**Figure 5:**
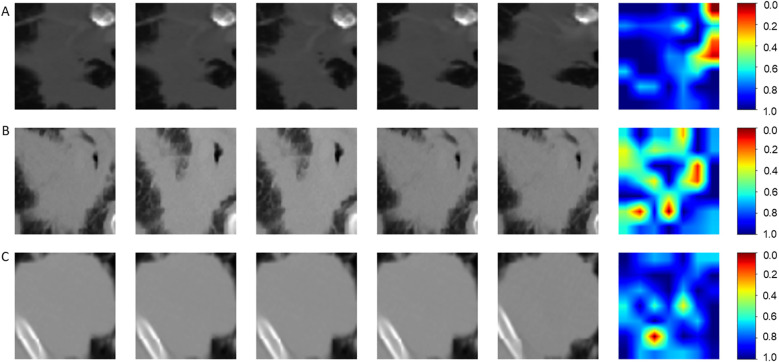
Grad-CAM-based qualitative interpretability analysis. Representative Grad-CAM maps from the 2D, 2.5D axial, and 3D models. Grad-CAM was used for qualitative visualization only and should not be interpreted as direct evidence of biological mechanism.

To qualitatively evaluate the spatial attention patterns of different model architectures, Grad-CAM maps were generated for a representative case using the 2D, 2.5D axial, and 3D models (**Figure 5**). Compared with the 2D model, which showed relatively scattered activation, and the 3D model, which demonstrated more extensive activation extending into surrounding non-tumor regions, the 2.5D axial model showed more concentrated activation within the tumor-containing region and tumor margin.

These findings provide a qualitative illustration of the spatial attention pattern of the 2.5D axial model and suggest that the model may rely more on tumor-centered imaging information than on unrelated background structures. Because the Grad-CAM analysis was performed for visual interpretation only, it should not be interpreted as direct evidence of a biological mechanism. Further population-level interpretability analyses and correlation with pathological or molecular data are needed to clarify the biological basis of the imaging patterns learned by the model.

## 4 Discussion

In this multicenter retrospective study, we developed and validated a CT-based 2.5D axial ResNet-101 model for predicting response to ICI-chemotherapy in patients with EGFR-mutant lung adenocarcinoma after first-generation EGFR-TKI resistance. The final analyzable cohort included 490 patients, comprising a training cohort and two independent validation cohorts. Among the evaluated 2D, 2.5D, and 3D architectures, the 2.5D axial model showed the most stable performance across cohorts, with AUCs of 0.885, 0.819, and 0.863 in the training cohort, validation cohort 1, and validation cohort 2, respectively. In addition to ORR prediction, the model-derived risk score also demonstrated significant PFS stratification across all three cohorts, supporting its potential value as a non-invasive imaging tool for treatment-response assessment and risk stratification in this clinically challenging population.

The clinical and methodological strengths of this study are fourfold. First, the study focused on a specific post-TKI resistance setting, namely patients with EGFR-mutant lung adenocarcinoma who had progressed on first-generation EGFR-TKIs without evidence of the T790M resistance mutation. Because third-generation EGFR-TKI therapy has been established for T790M-positive disease after prior EGFR-TKI progression, T790M-negative patients represent a clinically challenging subgroup with limited subsequent targeted options and an unmet need for reliable tools to guide ICI-chemotherapy selection (17). This population represents a clinically difficult subgroup with limited subsequent treatment options and an unmet need for reliable tools to guide ICI-chemotherapy selection. Second, the proposed model was based entirely on routine pretreatment CT images, providing a non-invasive imaging biomarker that may complement clinical decision-making when repeat tissue biopsy or comprehensive molecular profiling is impractical because of lesion accessibility, patient condition, sampling bias, or incomplete biomarker testing (18–20). Third, the 2.5D axial framework achieved a practical balance between spatial information and model complexity. By incorporating adjacent CT slices, the model captured more local tumor context than conventional single-slice 2D models, while avoiding the higher parameter burden and overfitting risk associated with fully volumetric 3D models in moderate-sized medical imaging datasets (10, 21, 22). Fourth, the use of two independent validation cohorts strengthened the assessment of model robustness and generalizability beyond the training cohort.

Compared with previous imaging studies in EGFR-mutant lung cancer, the present work addresses a different clinical question. Prior studies have largely focused on predicting EGFR mutation status or the T790M resistance mutation using radiomic features derived from medical images (23–25). In contrast, our model was designed to predict response to ICI-chemotherapy after EGFR-TKI resistance, a setting in which effective targeted options may be limited. From a methodological perspective, traditional radiomics approaches usually require predefined feature extraction and feature selection steps, which may introduce variability and dependence on handcrafted descriptors (26). The end-to-end deep learning framework used in this study may allow more direct learning of treatment-relevant imaging patterns from CT data, although the biological meaning of these learned features requires further validation.

The favorable performance of the 2.5D axial model may reflect its ability to capture subtle tumor-centered imaging phenotypes associated with treatment response. The Grad-CAM analysis provided a qualitative illustration that the 2.5D model tended to focus on tumor-containing regions and tumor margins rather than unrelated background structures. However, these visual findings should not be interpreted as direct evidence of a biological mechanism. We hypothesize that imaging patterns learned by the model may be related to tumor heterogeneity and treatment-associated microenvironmental changes during TKI resistance, such as altered immune contexture or angiogenic activity (27, 28). Nevertheless, this interpretation remains inferential. Future studies integrating matched imaging, histopathological, PD-L1, tumor mutational burden, immune infiltration, and genomic data are needed to clarify the biological basis of the CT phenotypes learned by the model. Decision curve analysis further suggested that the 2.5D axial model may provide potential clinical benefit across a range of threshold probabilities.

In terms of risk stratification, the model-derived risk score demonstrated significant PFS separation between high-risk and low-risk groups across the training cohort and both validation cohorts. These findings suggest that the imaging-derived response probability may provide supportive prognostic information regarding disease control after ICI-chemotherapy. Importantly, the survival analyses should be interpreted as supportive risk-stratification analyses rather than evidence that the model was directly trained for survival prediction. In the present study, the deep learning model was trained for ORR classification, whereas PFS was evaluated separately as a time-to-event outcome based on model-derived risk groups.

Several limitations should be acknowledged. First, this was a retrospective study from centers within one geographic region, and prospective validation in broader populations is required. Second, although two independent validation cohorts were included, external validation in cohorts from different healthcare systems and treatment contexts is still needed, particularly because some ICI regimens used in China may not be universally adopted in Western clinical practice. Third, PD-L1 expression, subsequent treatment information, and some clinical variables were not uniformly available across all centers, limiting standardized clinical-only and combined clinical-imaging model benchmarking. Therefore, the incremental value of the imaging model over a fully harmonized clinical model should be explored in future studies. Fourth, OS was not included in the survival analyses reported in this manuscript because it may be substantially confounded by subsequent lines of therapy after disease progression. Finally, Grad-CAM analysis was used only for qualitative visualization and does not establish a biological mechanism.

Based on these limitations, future studies should focus on prospective multicenter validation, standardized collection of clinical and molecular variables, and integrated modeling strategies combining CT-based deep learning features with clinical predictors, PD-L1 expression where available, genomic features, and treatment information. Such studies will be important to determine whether the imaging score provides incremental value over routine clinical assessment. In addition, matched radiological, pathological, and molecular analyses are needed to clarify the biological basis of the imaging phenotypes associated with response to ICI-chemotherapy.

In conclusion, this study developed and validated a CT-based 2.5D axial ResNet-101 model for non-invasive prediction of response to ICI-chemotherapy in EGFR-mutant lung adenocarcinoma after first-generation EGFR-TKI resistance. The model showed stable performance across the training cohort and two independent validation cohorts and provided supportive PFS risk stratification. These findings suggest that 2.5D CT-based deep learning may serve as a promising imaging tool for treatment-response assessment and individualized risk stratification in this clinically challenging setting.

## Data Availability

The original contributions presented in the study are included in the article/**Supplementary Material**. Further inquiries can be directed to the corresponding author.
