# Supplementary material for: Predicting response to immune checkpoint inhibitor plus chemotherapy in EGFR-mutant lung adenocarcinoma following first-generation TKI resistance: a multicenter deep learning study
Source: Front Immunol. 2026 Jul 17;17:1760264. doi: 10.3389/fimmu.2026.1760264 (PMC13423859; doi:10.3389/fimmu.2026.1760264)
Supplement: Supplementary file 1 [file DataSheet1.docx]

Predicting Response to Immune Checkpoint Inhibitor plus Chemotherapy in EGFR-Mutant Lung Adenocarcinoma following First-Generation TKI Resistance: A Multicenter Deep Learning Study

**Supplementary materials**

**Table S1.**

|  | ALL N=490 | Non-response N=323 | Response N=167 | P value |
| --- | --- | --- | --- | --- |
| **Sex** |  |  |  | 0.222 |
| Female | 306 (62.45%) | 195 (60.37%) | 111 (66.47%) |  |
| Male | 184 (37.55%) | 128 (39.63%) | 56 (33.53%) |  |
| **Age** | 62 (55-68) | 62 (55-69) | 62 (55-68) | 0.709 |
| **T stage** |  |  |  | 0.124 |
| T1 | 100 (20.41%) | 72 (22.29%) | 28 (16.77%) |  |
| T2 | 176 (35.92%) | 122 (37.77%) | 54 (32.34%) |  |
| T3 | 72 (14.69%) | 42 (13.00%) | 30 (17.96%) |  |
| T4 | 142 (28.98%) | 87 (26.93%) | 55 (32.93%) |  |
| **N stage** |  |  |  | 0.001 |
| N0 | 93 (18.98%) | 60 (18.58%) | 33 (19.76%) |  |
| N1 | 39 (7.96%) | 30 (9.29%) | 9 (5.39%) |  |
| N2 | 195 (39.80%) | 144 (44.58%) | 51 (30.54%) |  |
| N3 | 163 (33.27%) | 89 (27.55%) | 74 (44.31%) |  |
| **Chemotherapy**  **regimen** |  |  |  | 0.850 |
| Pemetrexed | 297 (60.61%) | 194 (60.06%) | 103 (61.68%) |  |
| Paclitaxel | 107 (21.84%) | 73 (22.60%) | 34 (20.36%) |  |
| Other | 86 (17.55%) | 56 (17.34%) | 30 (17.96%) |  |
| ECOG PS |  |  |  | 1.000 |
| 0-1 | 434 (88.57%) | 286 (88.54%) | 148 (88.62%) |  |
| 2 | 56 (11.43%) | 37 (11.46%) | 19 (11.38%) |  |
| **Primary site** |  |  |  | 0.807 |
| Left | 209 (42.65%) | 136 (42.11%) | 73 (43.71%) |  |
| Right | 281 (57.35%) | 187 (57.89%) | 94 (56.29%) |  |
| **Diabetes** |  |  |  | 0.010 |
| No | 422 (86.12%) | 288 (89.16%) | 134 (80.24%) |  |
| Yes | 68 (13.88%) | 35 (10.84%) | 33 (19.76%) |  |
| **Hypertension** |  |  |  | 0.446 |
| No | 307 (62.65%) | 198 (61.30%) | 109 (65.27%) |  |
| Yes | 183 (37.35%) | 125 (38.70%) | 58 (34.73%) |  |
| **Heart disease** |  |  |  | 0.092 |
| No | 381 (77.76%) | 259 (80.19%) | 122 (73.05%) |  |
| Yes | 109 (22.24%) | 64 (19.81%) | 45 (26.95%) |  |
| **Smoking** |  |  |  | 0.142 |
| No | 360 (73.47%) | 230 (71.21%) | 130 (77.84%) |  |
| Yes | 130 (26.53%) | 93 (28.79%) | 37 (22.16%) |  |
| **Drinking** |  |  |  | 0.764 |
| No | 214 (43.67%) | 139 (43.03%) | 75 (44.91%) |  |
| Yes | 276 (56.33%) | 184 (56.97%) | 92 (55.09%) |  |
| BMI | 23.90 (22.20;25.40) | 23.90 (22.35;25.20) | 23.90 (22.00;26.10) | 0.817 |
| **EGFR subtype** |  |  |  | 0.983 |
| 19 del | 186 (37.96%) | 122 (37.77%) | 64 (38.32%) |  |
| 21 L858R | 304 (62.04%) | 201 (62.23%) | 103 (61.68%) |  |
| **Brain metastasis** |  |  |  | 0.565 |
| No | 318 (64.90%) | 213 (65.94%) | 105 (62.87%) |  |
| Yes | 172 (35.10%) | 110 (34.06%) | 62 (37.13%) |  |
| **Liver metastasis** |  |  |  | 0.255 |
| No | 443 (90.41%) | 288 (89.16%) | 155 (92.81%) |  |
| Yes | 47 (9.59%) | 35 (10.84%) | 12 (7.19%) |  |
| **Bone metastasis** |  |  |  | 0.007 |
| No | 256 (52.24%) | 154 (47.68%) | 102 (61.08%) |  |
| Yes | 234 (47.76%) | 169 (52.32%) | 65 (38.92%) |  |
| **ICI** |  |  |  | 0.713 |
| **PD-1** | 17 (3.47%) | 10 (3.10%) | 7 (4.19%) |  |
| **PD-L1** | 473 (96.53%) | 313 (96.90%) | 160 (95.81%) |  |

**Table S2. Model reproducibility details.**

| **Item** | **Revised description** |
| --- | --- |
| **Final modeling cohort** | The final locked modeling cohort included 490 patients with complete eligible CT imaging and treatment response labels. The cohort was divided into a training cohort (n = 326), validation cohort 1 (n = 70), and validation cohort 2 (n = 94). |
| **Validation cohort independence** | Validation cohort 1 and validation cohort 2 were derived from institutionally independent centers and were used exclusively for independent external performance evaluation. |
| **Data leakage prevention** | No data from either validation cohort were used for model training, hyperparameter tuning, threshold selection, threshold derivation, or model selection. |
| **Classification endpoint** | The deep learning models were trained as binary classifiers for objective response after ICI-chemotherapy. Response-positive disease corresponded to complete response or partial response according to RECIST 1.1; response-negative disease corresponded to stable disease or progressive disease. |
| **Model output** | The model output represented the predicted probability of response-positive disease. For PFS stratification, the predicted response probability was converted into a risk score defined as 1 minus response probability, so that higher values indicated a higher predicted risk of non-response. |
| **2D input definition** | For the 2D models, tumor-centered CT images were extracted from three orthogonal views (axial, sagittal, and coronal). For each view, the slice with the largest tumor area was cropped around the ROI and resized to 256 x 256 pixels. |
| **2.5D definition** | The 2.5D approach was defined as a multi-channel pseudo-volumetric strategy in which five adjacent CT slices were stacked as input channels and processed by a 2D ResNet-101 backbone. |
| **2.5D input construction** | For each orthogonal view, the central slice with the largest tumor area and two adjacent slices on each side were selected at 1-mm intervals, resulting in five consecutive slices per view. The minimum bounding rectangle enclosing the tumor across the five slices was cropped and resized to 256 x 256 pixels. |
| **3D input definition** | For the 3D model, the tumor ROI was encapsulated within a bounding cube and resampled to 96 x 96 x 96 voxels before being used as volumetric input. |
| **Pretraining** | The 2D and 2.5D ResNet-101 backbones were initialized using ImageNet-pretrained weights and fine-tuned on the training cohort. The 3D ResNet-101 model was initialized from scratch because directly transferable ImageNet-pretrained weights are not readily available for standard volumetric convolutions. |
| **Data augmentation** | For the 2D and 2.5D models, data augmentation included random horizontal and vertical flipping and center cropping. For the 3D model, mirror flipping along the X, Y, and Z axes was applied. |
| **Loss function** | Binary cross-entropy loss was used for the ORR classification task. No survival-specific loss function or Cox proportional hazards layer was used in the deep learning models. |
| **Optimization and regularization** | Models were optimized using the Adam optimizer. The batch size was 64 for the 2D and 2.5D models, and dropout regularization was used to reduce overfitting. The 2.5D model used an initial learning rate of 0.001. |
| **Class imbalance handling** | In the training cohort, response-positive and response-negative cases were 118 and 208, respectively, indicating moderate class imbalance. No oversampling, undersampling, or class weighting strategy was applied. The class distribution was reported to support transparent interpretation of threshold-dependent performance metrics. |
| **Threshold selection** | For each model, the classification threshold was determined in the training cohort using the Youden index and then applied unchanged to validation cohort 1 and validation cohort 2. Thresholds were not re-optimized in either validation cohort. |
| **Performance metrics** | Model performance was evaluated using AUC, accuracy, sensitivity, specificity, PPV, NPV, and F1 score. Pairwise AUC comparisons were performed using DeLong tests with correction for multiple comparisons. |
| **Survival stratification** | PFS was evaluated separately as a time-to-event endpoint for risk stratification based on the 2.5D axial model output score. Overall survival was not included in the survival analyses reported in the manuscript. |
| **Segmentation reproducibility** | Tumor segmentation reproducibility was evaluated in the available 30-case subset. ICC values ranged from 0.973 to 1.000, with a median ICC of 0.998, supporting excellent interobserver reproducibility. |

**Abbreviations:** AUC, area under the curve; CT, computed tomography; ICI, immune checkpoint inhibitor; ICC, intraclass correlation coefficient; NPV, negative predictive value; ORR, objective response rate; PFS, progression-free survival; PPV, positive predictive value; RECIST, Response Evaluation Criteria in Solid Tumors; ROI, region of interest.

**Table S3.** **Performance of 2D models using training-derived thresholds.**

| Signature | Accuracy | AUC | 95% CI | Sensitivity | Specificity | PPV | NPV | F1 | Cohort |
| --- | --- | --- | --- | --- | --- | --- | --- | --- | --- |
| Axial | 0.718 | 0.724 | 0.667 - 0.782 | 0.765 | 0.655 | 0.749 | 0.674 | 0.757 | Training cohort |
| Coronal | 0.718 | 0.705 | 0.647 - 0.763 | 0.829 | 0.568 | 0.721 | 0.712 | 0.771 | Training cohort |
| Sagittal | 0.641 | 0.672 | 0.613 - 0.732 | 0.604 | 0.691 | 0.724 | 0.565 | 0.659 | Training cohort |
| Axial | 0.629 | 0.596 | 0.460 - 0.733 | 0.526 | 0.750 | 0.714 | 0.571 | 0.606 | Validation cohort 1 |
| Coronal | 0.543 | 0.526 | 0.389 - 0.664 | 0.237 | 0.906 | 0.750 | 0.500 | 0.360 | Validation cohort 1 |
| Sagittal | 0.600 | 0.622 | 0.489 - 0.754 | 0.526 | 0.687 | 0.667 | 0.550 | 0.588 | Validation cohort 1 |
| Axial | 0.713 | 0.705 | 0.594 - 0.815 | 0.511 | 0.898 | 0.821 | 0.667 | 0.630 | Validation cohort 2 |
| Coronal | 0.723 | 0.667 | 0.549 - 0.784 | 0.422 | 1.000 | 1.000 | 0.653 | 0.594 | Validation cohort 2 |
| Sagittal | 0.606 | 0.629 | 0.515 - 0.743 | 0.489 | 0.714 | 0.611 | 0.603 | 0.543 | Validation cohort 2 |

**Note.** AUC is a threshold-independent metric. Accuracy, sensitivity, specificity, PPV, NPV, and F1 score were calculated using model-specific thresholds derived from the training cohort by the Youden index and applied unchanged to validation cohort 1 and validation cohort 2. No threshold was re-optimized in either validation cohort.

**Table S4.** **Performance of 2.5D models using training-derived thresholds.**

| Signature | Accuracy | AUC | 95% CI | Sensitivity | Specificity | PPV | NPV | F1 | Cohort |
| --- | --- | --- | --- | --- | --- | --- | --- | --- | --- |
| Axial | 0.880 | 0.885 | 0.843 - 0.928 | 0.957 | 0.877 | 0.852 | 0.931 | 0.902 | Training cohort |
| Coronal | 0.764 | 0.737 | 0.679 - 0.796 | 0.914 | 0.561 | 0.737 | 0.83 | 0.816 | Training cohort |
| Sagittal | 0.709 | 0.715 | 0.658 - 0.772 | 0.845 | 0.525 | 0.705 | 0.716 | 0.769 | Training cohort |
| Axial | 0.814 | 0.819 | 0.709 - 0.943 | 0.947 | 0.856 | 0.766 | 0.913 | 0.847 | Validation cohort 1 |
| Coronal | 0.729 | 0.710 | 0.582 - 0.837 | 0.763 | 0.687 | 0.744 | 0.710 | 0.753 | Validation cohort 1 |
| Sagittal | 0.729 | 0.724 | 0.599 - 0.848 | 0.711 | 0.750 | 0.771 | 0.686 | 0.740 | Validation cohort 1 |
| Axial | 0.819 | 0.863 | 0.784 - 0.943 | 0.822 | 0.816 | 0.804 | 0.833 | 0.813 | Validation cohort 2 |
| Coronal | 0.755 | 0.760 | 0.661 - 0.859 | 0.822 | 0.694 | 0.712 | 0.810 | 0.763 | Validation cohort 2 |
| Sagittal | 0.734 | 0.718 | 0.609 - 0.827 | 0.844 | 0.633 | 0.679 | 0.816 | 0.752 | Validation cohort 2 |

**Note.** AUC is a threshold-independent metric. Accuracy, sensitivity, specificity, PPV, NPV, and F1 score were calculated using model-specific thresholds derived from the training cohort by the Youden index and applied unchanged to validation cohort 1 and validation cohort 2. No threshold was re-optimized in either validation cohort.

**Table S5.** **Performance of the 3D model using the training-derived threshold.**

| Signature | Accuracy | AUC | 95% CI | Sensitivity | Specificity | PPV | NPV | F1 | Cohort |
| --- | --- | --- | --- | --- | --- | --- | --- | --- | --- |
| 3D | 0.727 | 0.794 | 0.746 - 0.842 | 0.668 | 0.806 | 0.822 | 0.644 | 0.737 | Training cohort |
| 3D | 0.700 | 0.703 | 0.578 - 0.829 | 0.842 | 0.531 | 0.681 | 0.739 | 0.753 | Validation cohort 1 |
| 3D | 0.681 | 0.697 | 0.586 - 0.807 | 0.689 | 0.673 | 0.660 | 0.702 | 0.674 | Validation cohort 2 |

**Note.** AUC is a threshold-independent metric. Accuracy, sensitivity, specificity, PPV, NPV, and F1 score were calculated using model-specific thresholds derived from the training cohort by the Youden index and applied unchanged to validation cohort 1 and validation cohort 2. No threshold was re-optimized in either validation cohort.

**Table S6.** Interobserver reproducibility of tumor segmentation features in the available 30-case subset.

| **Feature** | **ICC** |
| --- | --- |
| original_shape_Sphericity | 0.9731 |
| original_shape_Elongation | 0.9854 |
| original_shape_Flatness | 0.9891 |
| original_shape_SurfaceVolumeRatio | 0.9917 |
| original_shape_LeastAxisLength | 0.9974 |
| original_shape_MinorAxisLength | 0.9976 |
| original_shape_SurfaceArea | 0.9980 |
| original_shape_MeshVolume | 0.9981 |
| original_shape_Maximum2DDiameterRow | 0.9981 |
| original_shape_Maximum3DDiameter | 0.9988 |
| original_shape_Maximum2DDiameterColumn | 0.9990 |
| original_shape_Maximum2DDiameterSlice | 0.9991 |
| original_shape_MajorAxisLength | 0.9995 |

**Note.** ICC, intraclass correlation coefficient. The available interobserver dataset contained 30 cases and 13 shape features. ICC values ranged from 0.9731 to 0.9995, with a median ICC of 0.9980, supporting excellent segmentation reproducibility.

**Table S7.** PFS Cox regression, training cohort.

|  | Univariate analysis | | | Multivariate analysis | | |
| --- | --- | --- | --- | --- | --- | --- |
| **characteristics** | HR | 95%CI | P | HR | 95%CI | P |
| **Age** | 1.002 | 0.989-1.014 | 0.81 |  |  |  |
| **BMI** | 1.095 | 0.925-1.296 | 0.291 |  |  |  |
| **Bone metastasis** |  |  |  |  |  |  |
| No | Reference |  |  |  |  |  |
| Yes | 1.244 | 0.971-1.594 | 0.084 |  |  |  |
| **Brain metastasis** |  |  |  |  |  |  |
| No | Reference |  |  |  |  |  |
| Yes | 0.917 | 0.708-1.188 | 0.512 |  |  |  |
| **Chemotherapy regimen** |  |  |  |  |  |  |
| Pemetrexed | Reference |  |  |  |  |  |
| Paclitaxel | 1.094 | 0.807-1.484 | 0.563 |  |  |  |
| Other | 0.97 | 0.683-1.376 | 0.864 |  |  |  |
| **Diabetes** |  |  |  |  |  |  |
| No | Reference |  |  |  |  |  |
| Yes | 0.805 | 0.569-1.138 | 0.219 |  |  |  |
| **Drinking** |  |  |  |  |  |  |
| No | Reference |  |  |  |  |  |
| Yes | 1.136 | 0.856-1.508 | 0.377 |  |  |  |
| **ECOG** |  |  |  |  |  |  |
| 0-1 | Reference |  |  |  |  |  |
| ≥2 | 0.911 | 0.614-1.351 | 0.644 |  |  |  |
| **EGFR subtype** |  |  |  |  |  |  |
| 19de | Reference |  |  |  |  |  |
| 21 L858R | 1.11 | 0.861-1.432 | 0.42 |  |  |  |
| **Heart disease** |  |  |  |  |  |  |
| No | Reference |  |  |  |  |  |
| Yes | 0.95 | 0.698-1.292 | 0.744 |  |  |  |
| **Hypertension** |  |  |  |  |  |  |
| No | Reference |  |  |  |  |  |
| Yes | 1.08 | 0.836-1.396 | 0.555 |  |  |  |
| **Liver metastasis** |  |  |  |  |  |  |
| No | Reference |  |  |  |  |  |
| Yes | 1.429 | 0.957-2.134 | 0.081 |  |  |  |
| **N stage** |  |  |  |  |  |  |
| N0 | Reference |  |  |  |  |  |
| N1 | 1.263 | 0.737-2.164 | 0.395 |  |  |  |
| N2 | 1.17 | 0.834-1.643 | 0.364 |  |  |  |
| N3 | 0.911 | 0.642-1.292 | 0.6 |  |  |  |
| **ICI** |  |  |  |  |  |  |
| PD-1 inhibitor | Reference |  |  |  |  |  |
| PD-L1 inhibitor | 1.183 | 0.607-2.305 | 0.621 |  |  |  |
| **Primary site** |  |  |  |  |  |  |
| Left | Reference |  |  |  |  |  |
| Right | 0.965 | 0.751-1.241 | 0.784 |  |  |  |
| **Risk** |  |  |  |  |  |  |
| Low risk | Reference |  |  |  |  |  |
| High risk | 1.637 | 1.255-2.136 | <0.001 | 1.708 | 1.305-2.234 | <0.001 |
| **Sex** |  |  |  |  |  |  |
| Female | Reference |  |  |  |  |  |
| Male | 1.106 | 0.854-1.434 | 0.444 |  |  |  |
| **Smoking** |  |  |  |  |  |  |
| No | Reference |  |  |  |  |  |
| Yes | 1.107 | 0.831-1.473 | 0.488 |  |  |  |
| **Stage** |  |  |  |  |  |  |
| IIIB | Reference |  |  |  |  |  |
| IV | 1.648 | 1.041-2.61 | 0.033 | 1.794 | 1.128-2.853 | 0.014 |
| **T stage** |  |  |  |  |  |  |
| T1 | Reference |  |  |  |  |  |
| T2 | 1.057 | 0.738-1.513 | 0.764 |  |  |  |
| T3 | 0.749 | 0.488-1.148 | 0.185 |  |  |  |
| T4 | 0.832 | 0.581-1.191 | 0.315 |  |  |  |

**Table S8.** PFS Cox regression, validation cohort 1.

|  | Univariate analysis | | | Multivariate analysis | | |
| --- | --- | --- | --- | --- | --- | --- |
| **characteristics** | HR | 95%CI | P | HR | 95%CI | P |
| **Age** | 0.983 | 0.955-1.011 | 0.231 |  |  |  |
| **BMI** | 1.027 | 0.743-1.417 | 0.874 |  |  |  |
| **Bone metastasis** |  |  |  |  |  |  |
| No | Reference |  |  |  |  |  |
| Yes | 1.513 | 0.912-2.512 | 0.109 |  |  |  |
| **Brain metastasis** |  |  |  |  |  |  |
| No | Reference |  |  |  |  |  |
| Yes | 1.095 | 0.646-1.855 | 0.737 |  |  |  |
| **Chemotherapy regimen** |  |  |  |  |  |  |
| Pemetrexed | Reference |  |  |  |  |  |
| Paclitaxel | 1.151 | 0.588-2.254 | 0.682 |  |  |  |
| Other | 0.753 | 0.38-1.493 | 0.417 |  |  |  |
| **Diabetes** |  |  |  |  |  |  |
| No | Reference |  |  |  |  |  |
| Yes | 1.121 | 0.565-2.225 | 0.744 |  |  |  |
| **Drinking** |  |  |  |  |  |  |
| No |  |  |  |  |  |  |
| Yes | 2.728 | 1.152-6.464 | 0.023 | 1.379 | 0.507-3.752 | 0.529 |
| **ECOG** |  |  |  |  |  |  |
| 0-1 |  |  |  |  |  |  |
| ≥2 | 1.294 | 0.464-3.609 | 0.622 |  |  |  |
| **EGFR subtype** |  |  |  |  |  |  |
| 19de | Reference |  |  |  |  |  |
| 21 L858R | 1.084 | 0.646-1.817 | 0.76 |  |  |  |
| **Heart disease** |  |  |  |  |  |  |
| No | Reference |  |  |  |  |  |
| Yes | 0.681 | 0.397-1.168 | 0.163 |  |  |  |
| **Hypertension** |  |  |  |  |  |  |
| No | Reference |  |  |  |  |  |
| Yes | 1.228 | 0.749-2.013 | 0.415 |  |  |  |
| **Liver metastasis** |  |  |  |  |  |  |
| No | Reference |  |  |  |  |  |
| Yes | 0.65 | 0.253-1.667 | 0.37 |  |  |  |
| **N stage** |  |  |  |  |  |  |
| N0 | Reference |  |  |  |  |  |
| N1 | 0.887 | 0.278-2.823 | 0.839 |  |  |  |
| N2 | 0.995 | 0.43-2.303 | 0.992 |  |  |  |
| N3 | 0.747 | 0.316-1.766 | 0.506 |  |  |  |
| **ICI** |  |  |  |  |  |  |
| PD-1 inhibitor | Reference |  |  |  |  |  |
| PD-L1 inhibitor | 0.364 | 0.087-1.52 | 0.166 |  |  |  |
| **Primary site** |  |  |  |  |  |  |
| Left | Reference |  |  |  |  |  |
| Right | 0.708 | 0.422-1.186 | 0.189 |  |  |  |
| **Risk** |  |  |  |  |  |  |
| Low risk | Reference |  |  |  |  |  |
| High risk | 1.963 | 1.144-3.368 | 0.014 | 1.926 | 1.117-3.32 | 0.018 |
| **Sex** |  |  |  |  |  |  |
| Female | Reference |  |  |  |  |  |
| Male | 2.668 | 1.538-4.629 | <0.001 | 1.909 | 0.837-4.354 | 0.124 |
| **Smoking** |  |  |  |  |  |  |
| No | Reference |  |  |  |  |  |
| Yes | 2.93 | 1.601-5.363 | <0.001 | 1.487 | 0.57-3.883 | 0.417 |
| **T stage** |  |  |  |  |  |  |
| T1 | Reference |  |  |  |  |  |
| T2 | 0.831 | 0.407-1.695 | 0.61 |  |  |  |
| T3 | 0.771 | 0.329-1.809 | 0.551 |  |  |  |
| T4 | 1.575 | 0.717-3.46 | 0.258 |  |  |  |
| **Stage** |  |  |  |  |  |  |
| IIIB | Reference |  |  |  |  |  |
| IV | 0.947 | 0.463-1.935 | 0.881 |  |  |  |

**Table S9.** PFS Cox regression, validation cohort 2.

|  | Univariate analysis | | | Multivariate analysis | | |
| --- | --- | --- | --- | --- | --- | --- |
| **characteristics** | HR | 95%CI | P | HR | 95%CI | P |
| **Age** | 0.987 | 0.963-1.011 | 0.289 |  |  |  |
| **BMI** | 0.959 | 0.665-1.381 | 0.821 |  |  |  |
| **Bone metastasis** |  |  |  |  |  |  |
| No | Reference |  |  |  |  |  |
| Yes | 1.313 | 0.828-2.081 | 0.247 |  |  |  |
| **Brain metastasis** |  |  |  |  |  |  |
| No | Reference |  |  |  |  |  |
| Yes | 1.249 | 0.782-1.995 | 0.351 |  |  |  |
| **Chemotherapy regimen** |  |  |  |  |  |  |
| Pemetrexed | Reference |  |  |  |  |  |
| Paclitaxel | 0.791 | 0.427-1.463 | 0.454 |  |  |  |
| Other | 0.776 | 0.417-1.445 | 0.424 |  |  |  |
| **Diabetes** |  |  |  |  |  |  |
| No | Reference |  |  |  |  |  |
| Yes | 0.593 | 0.256-1.371 | 0.222 |  |  |  |
| **Drinking** |  |  |  |  |  |  |
| No | Reference |  |  |  |  |  |
| Yes | 0.913 | 0.546-1.526 | 0.728 |  |  |  |
| **ECOG** |  |  |  |  |  |  |
| 0-1 | Reference |  |  |  |  |  |
| ≥2 | 0.825 | 0.389-1.751 | 0.617 |  |  |  |
| **EGFR subtype** |  |  |  |  |  |  |
| 19de | Reference |  |  |  |  |  |
| 21 L858R | 0.907 | 0.563-1.461 | 0.69 |  |  |  |
| **Heart disease** |  |  |  |  |  |  |
| No | Reference |  |  |  |  |  |
| Yes | 0.604 | 0.339-1.077 | 0.087 |  |  |  |
| **Hypertension** |  |  |  |  |  |  |
| No | Reference |  |  |  |  |  |
| Yes | 0.884 | 0.545-1.434 | 0.618 |  |  |  |
| **Liver metastasis** |  |  |  |  |  |  |
| **No** | Reference |  |  |  |  |  |
| **Yes** | 2.081 | 0.936-4.627 | 0.072 |  |  |  |
| **N stage** |  |  |  |  |  |  |
| N0 | Reference |  |  | Reference |  |  |
| N1 | 0.632 | 0.217-1.843 | 0.401 | 0.947 | 0.292-3.066 | 0.927 |
| N2 | 0.668 | 0.338-1.318 | 0.245 | 2.062 | 1.028-4.137 | 0.042 |
| N3 | 0.485 | 0.242-0.975 | 0.042 | 1.488 | 0.734-3.015 | 0.271 |
| **ICI** |  |  |  |  |  |  |
| PD-1 inhibitor | Reference |  |  | Reference |  |  |
| PD-L1 inhibitor | 0.359 | 0.129-0.998 | 0.049 | 1.483 | 0.51-4.311 | 0.469 |
| **Primary site** |  |  |  |  |  |  |
| Left | Reference |  |  |  |  |  |
| Right | 1.579 | 0.978-2.548 | 0.062 |  |  |  |
| **Risk** |  |  |  |  |  |  |
| Low risk | Reference |  |  | Reference |  |  |
| High risk | 5.798 | 3.023-11.121 | <0.001 | 2.533 | 1.52-4.222 | <0.001 |
| **Sex** |  |  |  |  |  |  |
| Female | Reference |  |  |  |  |  |
| Male | 0.927 | 0.585-1.47 | 0.747 |  |  |  |
| **Smoking** |  |  |  |  |  |  |
| No | Reference |  |  |  |  |  |
| Yes | 1.101 | 0.676-1.793 | 0.699 |  |  |  |
| **Stage** |  |  |  |  |  |  |
| IIIB | Reference |  |  |  |  |  |
| IV | 1.166 | 0.685-1.986 | 0.572 |  |  |  |
| **T stage** |  |  |  |  |  |  |
| T1 | Reference |  |  |  |  |  |
| T2 | 0.896 | 0.478-1.68 | 0.733 |  |  |  |
| T3 | 0.884 | 0.401-1.952 | 0.761 |  |  |  |
| T4 | 1.107 | 0.574-2.133 | 0.762 |  |  |  |
